# β-catenin/LEF1/IGF-IIR Signaling Axis Galvanizes the Angiotensin-II- induced Cardiac Hypertrophy

**DOI:** 10.3390/ijms20174288

**Published:** 2019-09-02

**Authors:** Chin-Hu Lai, Sudhir Pandey, Cecilia Hsuan Day, Tsung-Jung Ho, Ray-Jade Chen, Ruey-Lin Chang, Pei-Ying Pai, V. Vijaya Padma, Wei-Wen Kuo, Chih-Yang Huang

**Affiliations:** 1 Graduate Institute of Basic Medical Science, China Medical University, Taichung 404, Taiwan; 2 Division of Cardiovascular Surgery, Department of Surgery, Taichung Armed Force General Hospital, Taichung 411, Taiwan; 3 National Defense Medical Center, Taipei 114, Taiwan; 4 Graduate Institute of Biomedical Science, China Medical University, Taichung 404, Taiwan; 5 Department of Nursing, Mei Ho University, Pingguang Road, Pingtung 912, Taiwan; 6 Chinese Medicine, Hualien Tzu Chi Hospital, Buddhist Tzu Chi Medical Foundation, Tzu Chi University, Hualien 970, Taiwan; 7 Department of Surgery, School of Medicine, College of Medicine, Taipei Medical University, Taipei 110, Taiwan; 8 College of Chinese Medicine, School of Post-Baccalaureate Chinese Medicine, China Medical University, Taichung 404, Taiwan; 9 Division of Cardiology, China Medical University Hospital, Taichung 404, Taiwan; 10 Department of Biotechnology, Bharathiar University, Coimbatore 641046, India; 11 Department of Biological Science and Technology, China Medical University, Taichung 404, Taiwan; 12 Cardiovascular and Mitochondrial Related Disease Research Center, Hualien Tzu Chi Hospital, Buddhist Tzu Chi Medical Foundation, Hualien 970, Taiwan; 13 Center of General Education, Buddhist Tzu Chi Medical Foundation, Tzu Chi University of Science and Technology, Hualien 970, Taiwan; 14 Department of Medical Research, China Medical University Hospital, China Medical University, Taichung 404, Taiwan; 15 Department of Biotechnology, Asia University, Taichung 41354, Taiwan

**Keywords:** β-catenin, LEF1, Ang-II, IGF-IIR

## Abstract

Cardiovascular diseases have a high prevalence worldwide and constitute the leading causes of mortality. Recently, malfunctioning of β-catenin signaling has been addressed in hypertensive heart condition. Ang-II is an important mediator of cardiovascular remodeling processes which not only regulates blood pressure but also leads to pathological cardiac changes. However, the contribution of Ang-II/β-catenin axis in hypertrophied hearts is ill-defined. Employing in vitro H9c2 cells and in vivo spontaneously hypertensive rats (SHR) cardiac tissue samples, western blot analysis, luciferase assays, nuclear-cytosolic protein extracts, and immunoprecipitation assays, we found that under hypertensive condition β-catenin gets abnormally induced that co-activated LEF1 and lead to cardiac hypertrophy changes by up-regulating the IGF-IIR signaling pathway. We identified putative LEF1 consensus binding site on IGF-IIR promoter that could be regulated by β-catenin/LEF1 which in turn modulate the expression of cardiac hypertrophy agents. This study suggested that suppression of β-catenin expression under hypertensive condition could be exploited as a clinical strategy for cardiac pathological remodeling processes.

## 1. Introduction

Global prevalence of cardiovascular diseases has been on the rise and constitutes the leading causes of death worldwide [1]. Cardiac hypertrophy is an adaptive mechanism to enable un-interrupted healthier heart functions under stressful conditions. However, prolonged cardiac stresses may lead to irreversible cardiac hypertrophic responses that may result in the development of pathological cardiac structural and functional changes which may culminate into heart failure. Several causative factors have been suggested in pathological cardiac changes including angiotensin II (Ang-II), endothelin I, or catecholamines [2,3]. Ang-II is an important mediator of cardiovascular remodeling processes which not only increases blood pressure but also enhances the generation of reactive oxygen species (ROS) [4]. Our earlier studies have also shown the involvement of IGF-IIR signaling in the development of pathological cardiac changes during stresses via Gαq/PKCα/NFATC3 modulations [5,6]. Recently, β-catenin participation has been implicated in hypertensive heart condition [7]. β-catenin signaling displays dual and opposite effects on hematopoiesis and cardiac development, depending on the developmental stage. During the early stages of development, it promotes cardiogenesis and inhibits hematopoiesis, while later, it promotes blood cell formation and suppresses the cardiomyocyte differentiation [8]. Besides, adaptive cardiac remodeling upon chronic Ang-II challenge required β-catenin downregulation [9]. However, the role of Ang-II/β-catenin axis in cardiac hypertrophy responses is not completely elucidated. As such it is worthwhile to investigate the precise mechanisms of interplay between β-catenin/LEF1 signaling and Ang-II induced cardiac hypertrophy response. This study deciphered that β-catenin gets abnormally induced under hypertensive conditions which co-activated LEF1 and lead to cardiac hypertrophy changes by up-regulating the IGF-IIR signaling pathway.

## 2. Results

### 2.1. β-Catenin Expression Was Increased upon Ang-II Treatment in a Dose-Dependent Manner and Activated IGF-IIR/Gaq Mediated Cardiac Hypertrophy Pathway in H9c2 Cardiomyoblasts

**Figure 1:**
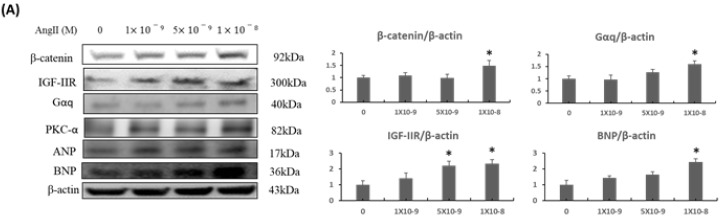

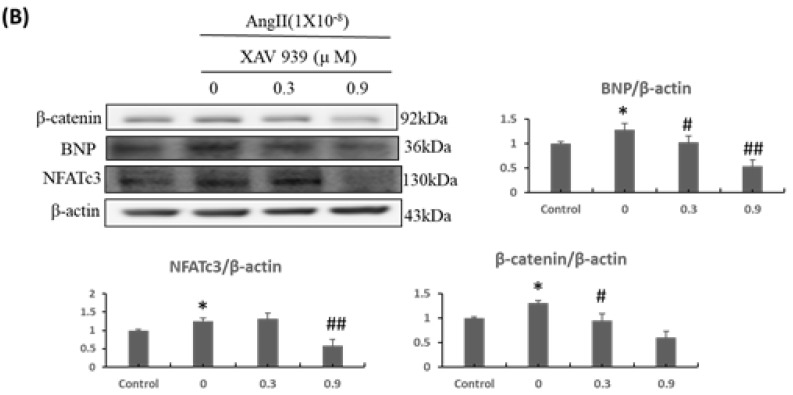
Effect of Ang-II treatment on endogenous β-catenin levels and hypertrophy associated protein markers in H9c2 cardiomyoblast cells. (**A**) H9c2 cells were treated with increasing concentrations of Ang-II and incubated for 24 h. At the end of the incubation period, cells were harvested, protein extracted, and subjected to western blot (WB) analysis. β-catenin and IGF-IIR expression were assessed along with downstream cardiac hypertrophy associated protein markers. (**B**) Following above experiment, 100 nM Ang-II treatments were selected for further experimental usage. H9c2 cells were subjected to Ang-II treatment with incubation for 24 h, followed by treatment with β-catenin inhibitor XAV 939 at increasing concentrations (0.3 µM, 0.9 µM, respectively) for another 12 h. At the end of 36 h, cells were harvested, protein extracted, and WB analysis was performed. Fold with respect to untreated controls have been shown in graphs. *p* * < 0.05, # < 0.05, ## < 0.01.

To elucidate the contribution of β-catenin/LEF1 signaling axis in cardiac hypertrophy, Ang-II treatment was used as a stimulus in a dose-dependent manner as it is known to induce hypertrophic responses under cardiac stresses. We found that Ang-II treatment could induce protein expressions of IGF-IIR, Gαq, PKC-α, and cardiac hypertrophy markers ANP, BNP. Interestingly, the β-catenin expression was increased upon Ang-II treatment in a dose-dependent manner (Figure 1A). However, when β-catenin expression was suppressed using an inhibitor XAV 939, cardiac hypertrophy associated transcription factor NFATc3 and hypertrophy marker BNP were also downregulated (Figure 1B).

### 2.2. β-Catenin Alterations Modulated the Cardiac Hypertrophy Associated Signaling Effectors both In Vitro and In Vivo

**Figure 2:**
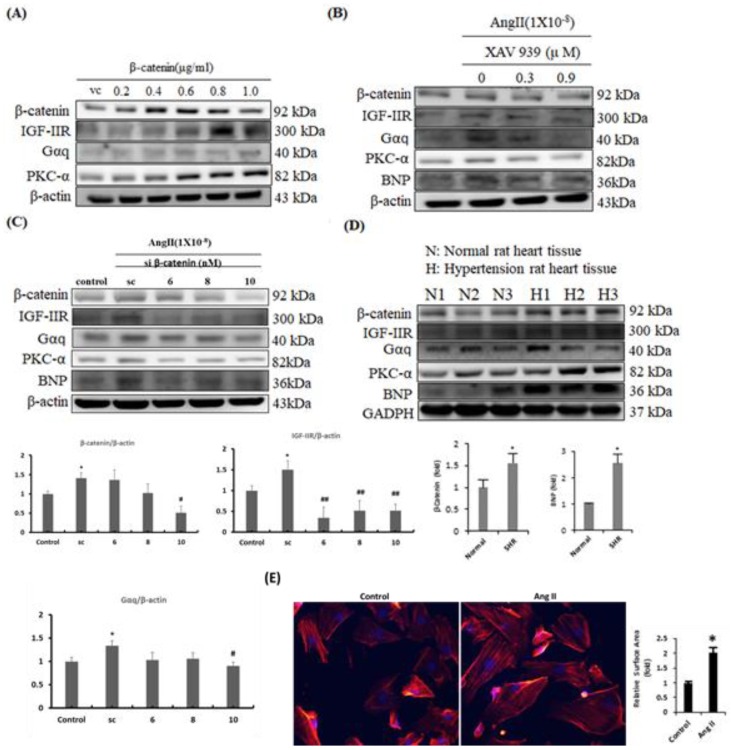
Effect of increased β-catenin and/or inhibition on IGF-IIR signaling in H9c2 cells and in hypertensive cardiac tissues. (**A**) In H9c2 cells, β–catenin plasmids were transfected in a dose-dependent manner and incubated for 24 h. At the end of the incubation period, cells were harvested and subjected to WB analysis. (**B**) WB analysis showing protein expression of β–catenin, IGF-IIR, Gαq, PKC-α, and BNP upon Ang-II and XAV 939 treatment as done previously. (**C**) H9c2 cells were treated with 100 nM Ang-II and incubated for 24 h. Following 24 h, si-RNA targeting β–catenin was transfected at increasing concentrations (scrambled (sc), 6 nM, 8 nM, 10 nM) for another 24 h. At the end of 48 h, cells were harvested, protein extracted, and analyzed for proteins described above by WB analysis. (**D**) WB analysis showing protein expressions of β–catenin, IGF-IIR and cardiac hypertrophy associated markers in cardiac tissues from SHRs. (**E**) Hypertrophy was assessed in H9c2 cells upon Ang-II treatment for 24 h using Actin-phalloidin staining. *n* = 3, *p* * < 0.05, *p* # < 0.05, *p* ## < 0.01.

To further confirm the contribution of β-catenin in hypertrophied hearts, gain-of-function, and loss-of-function approaches were utilized in H9c2 cells. Upon β-catenin overexpression, IGF-IIR, Gαq, and PKC-α were upregulated dose-dependently (Figure 2A). However, when β-catenin was suppressed using chemical inhibitor XAV 939 or si-RNA, in combination with Ang-II treatment, the expression of IGF-IIR, Gαq, PKC-α, and BNP were downregulated dose-dependently (Figure 2B,C). Next, we tested cardiac tissues from SHRs for β-catenin expression. We found that β-catenin expression was enhanced in myocardial tissues from SHRs that correlated with protein expressions of IGF-IIR, Gαq, PKC-α, and BNP (Figure 2D). Our earlier studies have shown that IGF-IIR signaling under hypertension conditions could lead to development of cardiac hypertrophy changes. Therefore, we also assessed the hypertrophic effect in H9c2 cardiomyocytes upon Ang-II treatment by immunofluorescence staining for actin filaments (Figure 2E). The results were in concordance with our earlier reports and showed that Ang-II treatment significantly increased the H9c2 cell surface area as compared to controls. Together, these data indicate that β-catenin contributes to cardiomyocyte hypertrophy responses during cardiac stresses.

### 2.3. Ang-II Treatment Enhanced Nuclear Enrichment of β-catenin, GATA-4 and NFATc3 in H9c2 Cardiomyoblast Cells

**Figure 3:**
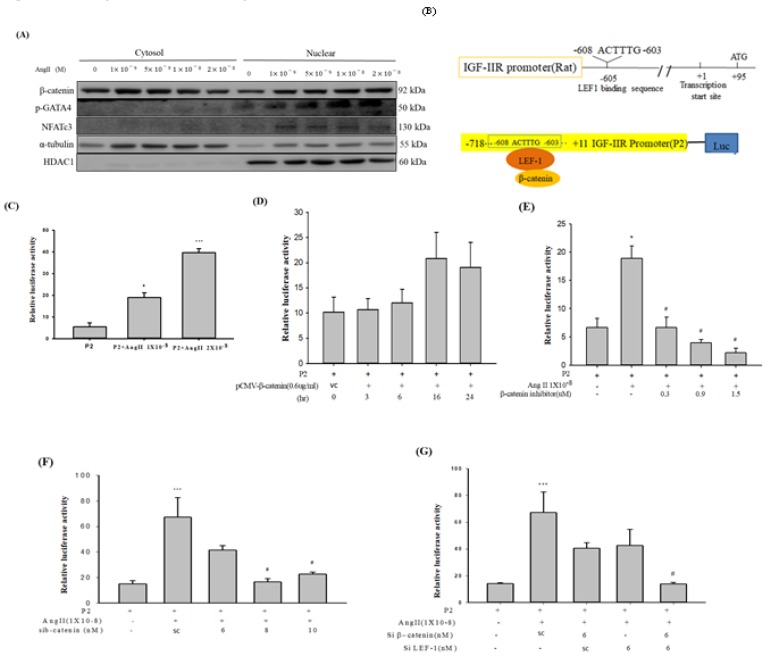
Effect of Ang-II treatment on nuclear enrichment of cardiac hypertrophy associated transcription factors and IGF-IIR promoter activity. (**A**) H9c2 cells were exposed to increasing concentrations of Ang-II and incubated for 48 h. At the end of the incubation period, cytosolic and nuclear protein extracts were prepared and subjected to western blot (WB) analysis. Protein levels of β-catenin, p-GATA4 and NFATc3 were measured in nuclear and cytosolic protein fractions. (**B**) Schematic representation of IGF-IIR promoter luciferase reporter constructs. (**C**) H9c2 cells were transfected with IGF-IIR luciferase reporter and incubated for 12 h. Next, they were exposed to increasing concentrations of Ang-II for another 24 h. At the end of 36 h, cell lysates were collected, and luciferase assay was performed. (**D**) IGF-IIR luciferase reporter constructs were transfected into H9c2 cells as before and incubated for 12 h. Next, they were transfected with β-catenin overexpression plasmid (0.6 µg/mL) and incubated for varying time points (3 h, 6 h, 16 h, 24 h). At the end of each time point, cell lysates were collected, and luciferase assays were performed. (**E**) H9c2 cells were transfected with IGF-IIR luciferase reporter construct as before and incubated for 12 h. Next, they were exposed to Ang-II treatment (100 nM) for another 24 h. Next, they were subjected to β-catenin inhibitor treatment (XAV 939, 0.3 µM 0.9 µM, 1.5 µM, respectively) for 12 h. At the end of 48 h, cell lysates were collected, and luciferase assays were performed. (**F**) IGF-IIR luciferase reporter plasmids were transfected into H9c2 cells as before and incubated for 12 h. Subsequently, they were treated with Ang-II (100 nM) for another 24 h. Next, they were subjected to β-catenin si-RNA treatment at increasing concentrations (scrambled (sc), 6 nM, 8 nM, 10 nM, respectively) for another 24 h. Following end of treatments, cell lysates were collected, and luciferase assays were performed. (**G**) IGF-IIR luciferase reporter plasmids were transfected into H9c2 cells as before and incubated for 12 h. Subsequently, they were treated with Ang-II (100 nM) for another 24 h. Next, they were co-transfected with β-catenin and/or LEF1 si-RNAs (scrambled (sc), 6 nM, respectively), incubated for another 24 h. At the of the end treatments, cell lysates were collected, and luciferase assays were performed. *p* * < 0.05, *p* *** < 0.01, *p* ^#^ < 0.05.

Previous researches have implicated GATA-4 and NFATc3 as primary transcription factors in cardiac hypertrophy responses. Therefore, we pursued to determine the effect of Ang-II treatment on nucleo-cytoplasmic enrichment of β-catenin. Our results indicated that Ang-II treatment in a dose-dependent manner induced nuclear mobilization of β-catenin co-enriched with GATA-4 and NFATc3. These data indicate that β-catenin has a partial contribution to Ang-II induced hypertrophy responses during cardiac stresses (Figure 3A).

### 2.4. IGF-IIR Promoter was Regulated by β-catenin and LEF1

β-catenin is known to act as a transcriptional co-activator of TCF/LEF1 target genes. Since β-catenin nuclear translocation of was increased by Ang-II, we questioned whether β-catenin could modulate IGF-IIR transcription via β-catenin/LEF1. A conserved LEF1 binding sequence 603 bp upstream of the ATG transcription start site was identified on IGF-IIR promoter using bioinformatics analysis (Figure 3B). Next, using luciferase reporter assays, we assessed whether Ang-II treatment could modulate the IGF-IIR promoter activity via β-catenin/LEF1. IGF-IIR promoter activity was enhanced upon Ang-II treatment in a dose-dependent as compared to controls (Figure 3C). This observation was further confirmed by overexpressing β-catenin and co-transfection with IGF-IIR luciferase reporter construct. As observed with Ang-II treatment, β-catenin overexpression also elicited similarly increased IGF-IIR promoter activity in a time-dependent course in H9c2 cells (Figure 3D). However, when β-catenin was inhibited using chemical inhibitor XAV 939 (Figure 3E) or si-RNA (Figure 3F), IGF-IIR luciferase promoter activity due to Ang-II treatment was suppressed in a dose-dependent manner. Interestingly, when both β-catenin and/or LEF1 were silenced together, it led to cumulative inhibition of Ang-II induced IGF-IIR promoter activity (Figure 3G). Together, these data suggest that β-catenin, in-conjunction with LEF1, modulates the IGF-IIR promoter through a preserved binding element for LEF1.

### 2.5. Inhibition of β-Catenin and/or LEF1 Alleviated the Hypertrophic Effect induced by Ang-II Treatment via Suppressing IGF-IIR/Gαq/PKCα Pathway

**Figure 4:**
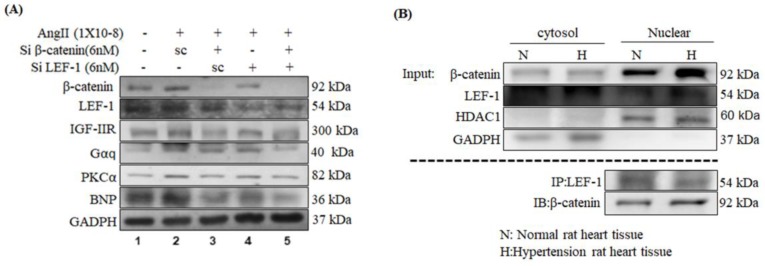
Effect of knockdown of β-catenin and/or LEF-1 on IGF-IIR pathway in H9c2 cells. (**A**) H9c2 cells were treated with Ang-II (100 nM) and incubated for 24 h. Next, they were transfected with si-RNAs (6 nM) against β-catenin, or LEF1, or both, and incubated for another 24 h. At the end of 48 h, cellular proteins were extracted, and WB analysis was performed to assess the levels of IGF-IIR, Gαq, PKC-α, and BNP upon silencing either β–catenin, or LEF1, or both. Fold with respect to untreated controls have been shown in graphs. (**B**) From the hypertensive cardiac tissues, nuclear, and cytosolic protein fractions were extracted and subjected to immunoprecipitation analysis using LEF1 antibodies. Subsequently, WB analysis was performed to assess β–catenin levels in above immunoprecipated proteins using β–catenin specific antibody. The results showed β-catenin protein level was increased in the nuclear fraction of hypertension rat tissue and directly bound to LEF-1. These results suggested that Ang-II induced cardiac hypertrophy responses involve β-catenin induced IGF-IIR upregulation and activation of downstream hypertrophic agents Gαq, PKC-α, ANP, and BNP.

To further assure the function of β-catenin/LEF1 in Ang-II-induced hypertrophy, either β-catenin or LEF1 or both were silenced using si-RNA with/without Ang-II treatment. Protein expression analysis revealed Ang-II induced cardiac hypertrophy agents such as IGF-IIR, Gαq, and PKCα, were downregulated upon silencing of either β-catenin, or LEF1, or both (Figure 4A).

Further, immunoprecipitation and protein expression analysis in the SHRs revealed β-catenin protein level was increased in the nuclear fraction of hypertensive myocardial tissues and directly bound to LEF1 (Figure 4B).

## 3. Methodology

### 3.1. Cell Culture

H9c2 cells were procured from American Type Culture Collection (ATCC, USA) and grown in DMEM medium supplied with 10% fetal bovine serum, glutamine (2 mM), penicillin (100 U/mL), streptomycin (100 mg/mL), and pyruvate (1 mM) in humidified air (5% CO_2_) at 37 °C. H9c2 cells at 60–70% confluence were used for experimental treatments. pCMV-β-catenin plasmid or siRNAs against β-catenin or LEF1 (MDBio, Taipei, Taiwan) were delivered into the cells using PureFection^TM^ Reagent (System Biosciences, Palo Alto, CA, USA) as per the instruction manual. XAV939 (β-catenin signaling inhibitor) (Sigma-Aldrich, X3004, USA) was used at varying concentrations and time durations as indicated.

### 3.2. Animal Model

Spontaneously hypertensive rats (SHR) 8-week-old (approx. 200 g), were acquired from the National Laboratory Animal Center (Taipei, Taiwan) and sustained following our previous protocol [10]. All animal experiments were performed in accordance with the Guide for the Care and Use of Laboratory Animals (National Institutes of Health Publication No. 85-23, revised 1996) under a protocol (Approval No. 2016-065-1, valid from 8/2016 to 7/2017) approved by the Animal Research Committee of China Medical University, Taichung, Taiwan. SHR animals with over 180/80 mmHg blood pressure were randomly selected, and hearts were collected after sacrifice for further experiments.

### 3.3. Western Blotting

Cellular protein extracts were prepared following previous publication with minor modifications as described [11]. Lysis buffer containing Tris-base (pH 7.4, 50 mM/L), NaCl (0.5 M/L), EDTA (1 M/L), NP-40 (1%), beta-mercaptoethanol (1 mM/L), IGEPAL CA-630 (Sigma-Aldrich), 10% glycerol, and protease inhibitors (Roche). The supernatants were collected after centrifugation at 16,000 rpm for 30 min, and protein amounts were estimated using Bradford assay (Bio-Rad, USA). SDS-PAGE (8%–12%) was used to resolve protein samples and then, blotted onto PVDF membranes (Millipore). Blocking was done using 5% skimmed milk powder (Tris-buffered saline-Tween-20) for 1 h. The membranes were incubated with diluted primary antibodies (1:1000) at 4 °C overnight followed by secondary antibodies incubation at RT for 1 h. The following antibodies were used: IGF-IIR (Abcam, Cat. No. ab124767), β-Catenin (C-18) (sc-1496), LEF-1(H-70) (sc-28687), Gαq (sc-392), p-GATA4 (sc-32823), NFATc3 (sc8405), p-PKCα (Upstate, Cat. No. 06-822), ANP (FL-153) (sc-20158), BNP (sc-18818), HDAC1 (sc-7872), α-Tubulin (B-7) (sc-5286), GAPDH (6C5) (sc-32233), and β-actin (sc-47778). Protein bands were visualized using enhanced chemiluminescence (ECL) horseradish peroxidase (HRP) substrate (Millipore) and densitometry analysis was performed using a LAS-3000 fluorescence imaging system (Fuji Film, Tokyo, Japan).

### 3.4. Actin Staining

H9c2 cells were fixed with 4% paraformaldehyde at room temperature (RT) for 30 min, washed with ice-cold PBS and permeabilized with 0.1% Triton X-100 for 10 min at 4 °C. Blocking was done in PBS containing 2% BSA at RT for 30 min. Actin filaments were stained using rhodamine- phalloidin and images were captured under a fluorescence microscope (magnification X 200). Cell surface area was determined using Image J software (NIH).

### 3.5. Immunoprecipitations (IP)

PureProteome Protein G Magnetic Bead System (Millipore) was used for IP as per instruction manual. 300 µg of protein extracts were used for processing and next, incubated with 2 μg of LEF1 antibody. Next, to each IP reaction, Protein G Magnetic Bead (50 µL) was added and then incubated overnight at 4 °C on a rotor. Elution was done at 95 °C for 5 min and then separated by SDS-PAGE. After transferring proteins to PVDF membrane, they were probed with specific primary antibodies.

### 3.6. Luciferase Assay

Dual-Glo Luciferase Assay (Promega, Sunnyvale, CA, USA) was used to assess IGF-IIR promoter activity in H9c2 cells. Briefly, cell lysates at the end of various treatments were collected and luminescence was detected by Luminometer (Turner Biosystems, Sunnyvale, CA, USA).

### 3.7. Nuclear Protein Extraction

Cytosolic and nuclear protein extracts were prepared using the Nuclear/Cytosol Fractionation Kit (BioVision, Milpitas, CA, USA) as per instruction manual. Isolated proteins were quantified using Bradford assay (Bio-Rad), and subjected to further analysis.

### 3.8. Statistical Analysis

Student’s t-test was used to assess the significance using SPSS10.0 software and a *p*-value < 0.05 was considered to be statistically significant. The data shown represent mean ± SD from at least three experimental replicates.

## 4. Discussion

Through this study, we examined the impact of β-catenin in Ang-II induced hypertrophy responses and demonstrated that IGF-IIR expression could be regulated by β-catenin/LEF1 leading to pathological cardiac hypertrophy signaling. Our earlier studies have shown that abnormal activation of IGF-IIR signaling pathway during cardiac stresses such as elevated Ang II levels [3], or high glucose [5], or drug-induced (doxorubicin) cardiotoxicity [12,13] could lead to increased hypertrophy, fibrosis, and cardiac apoptosis paving the way for heart failure. Besides, we showed that enhancement of β-catenin in cardiomyocytes promotes apoptosis and fibrosis during cardiac stresses such as myocardial infarction (MI) or diabetes [14]. This study showed that Ang-II treatment could upregulate β-catenin expression in parallel with IGF-IIR expression in a dose-dependent assessment. The downstream implication of the above effect resulted in the activation of a hypertrophic cascade mediated via Gαq/PKCα/NFATc3. However, when β-catenin was inhibited, the hypertrophic effects induced by Ang-II were ameliorated. Next, when β-catenin was overexpressed exogenously, it leads to increased expression of IGF-IIR. Conversely, inhibition of β-catenin using chemical inhibitor or si-RNA, in a dose-dependent manner, suppressed Ang-II instigated hypertrophy associated agents such as Gαq/PKCα/BNP. We further tested the protein expression level of those markers in cardiac tissues from hypertensive rats that also presented with similar results. However, several other studies [15,16] have implicated the transcription factors NFATc3 and GATA4 in development of pathological cardiac hypertrophy. Therefore, we also deduced the nuclear enrichment of cardiac hypertrophy associated transcription factors and noticed there was increased nuclear enrichment of GATA4 and NFATc3 with β-catenin upon Ang-II treatment in a dose-dependent assessment. These indications along with morphologically observed increased cardiomyocyte size suggested the contribution of β-catenin in Ang-II induced pathological cardiac hypertrophy via IGF-IIR. Several studies have shown β-catenin translocate from the cytosol into the nucleus where it conjugates with T-cell factor (TCF) and LEF family members to modulate genes possessing TCF/LEF1 binding sequences in their promoters [17,18,19]. This process has also been associated with the development of concentric hypertrophy in cardiomyocytes, arrhythmogenic cardiomyopathy, and mediates hypertensive heart diseases [20,21,22]. These elucidations propelled us to ask whether IGF-IIR could be a target of β-catenin/LEF1. When compared to controls, β-catenin protein level was increased in the nuclear fraction of hypertension rat cardiac tissue and directly bound to LEF1. Our results suggested that transcription of IGF-IIR during Ang-II induced hypertrophy is directly regulated via β-catenin through a preserved binding site for LEF1 on its promoter. Ang-II treatment could increase luciferase reporter based IGF-IIR promoter activity in a dose-dependent course. Interestingly, β-catenin overexpression also elicited a similar observation as above enhancing the IGF-IIR promoter activity. However, knockdown of β-catenin decreased IGF-IIR promoter activity, hence endorsing the contribution of β-catenin in Ang-II induced hypertrophy in the heart via IGF-IIR. Further, silencing of β-catenin and LEF1 leads to synergistic suppression of IGF-IIR promoter activity upon Ang-II treatment. This attenuation of IGF-IIR promoter activity upon inhibition of β-catenin was accompanied with decreased cardiac hypertrophy promoting agents such as Gαq, PKCα, and BNP.

In summary, Ang-II induced cardiac hypertrophy responses to involve partial contribution through increased β-catenin expression which, in turn, upregulate IGF-IIR expression via a consensus TCF/LEF1 binding sequence on IGF-IIR promoter and, thereafter, IGF-IIR signaling activates the downstream cardiac hypertrophy cascade.
